# Modelling Visual Change Detection and Identification under Free Viewing Conditions

**DOI:** 10.1371/journal.pone.0149217

**Published:** 2016-02-16

**Authors:** Ken McAnally, Russell Martin

**Affiliations:** Aerospace Division, Defence Science and Technology Group, Melbourne, Victoria, Australia; University of Melbourne, AUSTRALIA

## Abstract

We examined whether the abilities of observers to perform an analogue of a real-world monitoring task involving detection and identification of changes to items in a visual display could be explained better by models based on signal detection theory (SDT) or high threshold theory (HTT). Our study differed from most previous studies in that observers were allowed to inspect the initial display for 3s, simulating the long inspection times typical of natural viewing, and their eye movements were not constrained. For the majority of observers, combined change detection and identification performance was best modelled by a SDT-based process that assumed that memory resources were distributed across all eight items in our displays. Some observers required a parameter to allow for sometimes making random guesses at the identities of changes they had missed. However, the performance of a small proportion of observers was best explained by a HTT-based model that allowed for lapses of attention.

## Introduction

Operators in many work environments (e.g., air traffic control) are required to monitor and detect changes in visual displays. Where such changes are not signalled by transients, their detection depends in part on the operator’s ability to encode the information presented in the display and retain it in memory until the display can be resampled.

In laboratory settings, change-detection paradigms have commonly been used to study the architecture and capacity of visual short-term memory (VSTM). It is well established that VSTM is capacity limited but the literature is divided with regard to the nature of the limitation (see [1] for a review). Some models of VSTM postulate that it is limited in the number of discrete representations it can hold to around three or four "slots" [2–6]. Other models suggest that VSTM is a limited but continuous resource that can be distributed across a large, and perhaps unlimited, number of representations. As the number of representations is increased, the amount of resource available for each is reduced, resulting in less precise representations [7–9].

As most studies of VSTM have employed very short display inspection times (typically 100 to 500 ms), it is not clear whether these models will translate well to real-world settings where display inspection times are of the order of a few seconds and eye movements are not constrained. Longer inspection times will allow more eye movements during the inspection period and increase the probability that visual information is recoded and stored in a more durable form, e.g., as verbal labels. Where such recoding occurs, human performance may be better modelled by robust, noiseless representations than by noisy ones.

We examined the detection of changes in displays modelled on military tactical displays that were viewed for three seconds. We compared discrete-resource memory models based on high threshold theory (HTT) and continuous-resource models based on signal detection theory (SDT). We included a contemporary variant of HTT which allows for lapses of attention [5] and a variant in which limitations in the precision of memory representations [10] result in imperfect change detection for stimuli held in memory. We then examined whether these models could parsimoniously describe both change detection and change identification performance. The abilities to detect and identify changes are both of relevance in many real-world settings. For example, it is not normally sufficient for an air traffic controller to detect a change to his/her display—he/she must also be able to identify the changed symbol. Requiring models to fit change identification data in addition to change detection data provides a stronger test of their relative validities.

## Methods

### Participants

Ten observers (6 male, 4 female) participated in this study. Their average age was 22.1 (s.d. = 1.4) years. Eight had normal vision and two had corrected-to-normal vision. All gave informed written consent. The project was given ethics approval (number AOD 02–09) by the Chief of Air Operations Division, Defence Science and Technology Organisation, in accordance with the National Statement on Ethical Conduct in Human Research [11].

### Task

**Fig 1:**
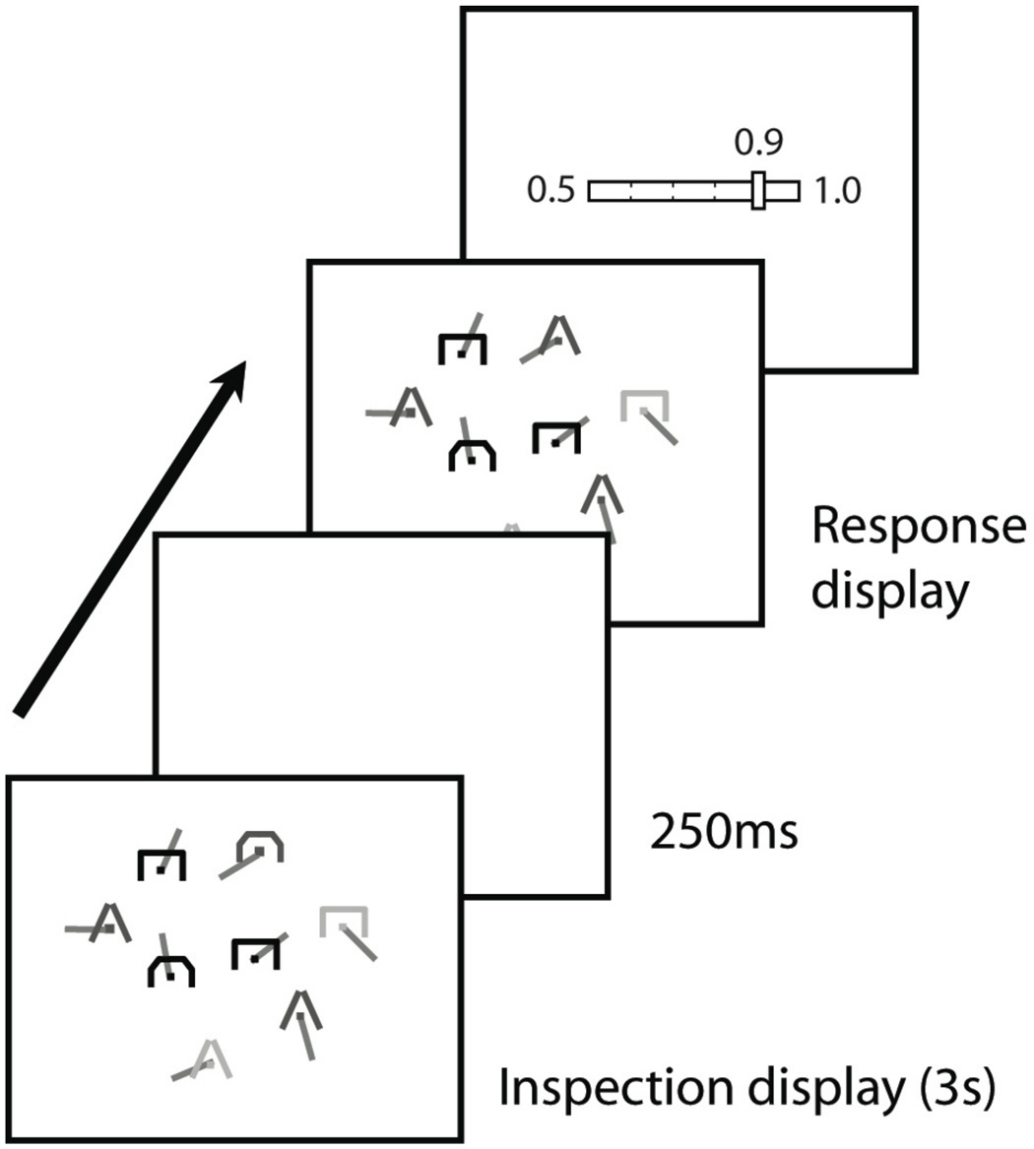
Time course of a trial. There were eight randomly placed symbols. Each symbol was of one of three shapes and one of three colours, and had a random line orientation. On a proportion (.25 or .75) of trials, the shape of one symbol changed between the first and second displays. Observers made a yes/no change detection response and then rated their confidence in that response using five confidence bins ranging from guess (.5 correct) to certain (1.0 correct). Following the confidence rating, observers indicated with a mouse the symbol that they believed was most likely to have changed.

Observers performed a change detection task for symbol shape while viewing displays modelled on military tactical displays in each of which eight symbols from the Hostile, Ambiguous, Friendly, Unknown (HAFU) symbol set [12] were presented (Fig 1). Observers were naive with respect to the semantic content of these symbols. Each symbol subtended approximately 0.6° in height and width and had a white line of length 1.3° emanating from its centre in a random direction. The location of each symbol was selected at random with the constraint that symbols did not overlap. Both symbol shape and symbol colour were selected at random with replacement from a set of three (open triangle, open rectangle, open semi-circle and blue, red, yellow, respectively). On each trial, an initial display was presented for 3 seconds and followed by a uniform grey mask of 0.25-second duration. A second display was then presented until the observer responded with his/her detection, confidence and identification decisions (see below). The second display was identical to the first except that on a proportion of trials (either .25 or .75) the shape of one randomly selected symbol had been changed to another in the set. Displays were presented on a 24" LCD monitor (Hewlett Packard 2465) at a viewing distance of approximately 60 cm.

Data were collected in 12 blocks of 40 trials. The two conditions of change probability (.25 and .75) were presented in separate blocks. The order of presentation of these conditions was counterbalanced within and across observers. Observers were naive with respect to the probabilities of change.

The observers' task was to indicate with a mouse whether or not one of the symbols had changed shape between presentations of the first and second displays. Observers then indicated their levels of confidence in their change detection decisions on a scale that was divided into 5 segments ranging from guess to certain. These confidence ratings were used to generate 9-point receiver operating characteristics (ROCs, following [13]). Observers were then asked to indicate with the mouse the item they believed was most likely to have changed. This change identification decision was made for all trials, including those where observers indicated they thought a change had not occurred. No feedback was given in regard to the accuracy of detection or identification responses.

### Models tested

#### Noiseless slots (HTT)

This model is based on HTT and incorporates a limited number of noiseless memory representations. According to this model, when the number of items (*N*) exceeds the capacity of memory (*k*), only a proportion (*k*/*N*) of items is represented in memory. A change is correctly detected (i.e., a hit occurs) if, and only if, either of the following two mutually exclusive events occurs: (i) the changed item is held in memory or (ii) the changed item is not held in memory but the observer guesses that there was a change. An observer guesses that there was a change when a changed item is not held in memory at his/her false-alarm rate. The hit rate (*H*) is therefore given by

$$H=\frac{k}{N}+\left(1-\frac{k}{N}\right)F$$

where *F* is the false-alarm rate.

Where a hit occurs, the changed item is correctly identified when it is held in memory. When it is not held in memory, its identity is guessed at from the set of items not held in memory. The correct identification rate for hits (*HID*) is therefore given by

$$HID=\frac{1}{H}\left[\frac{k}{N}+\frac{\left(1-\frac{k}{N}\right)F}{N-k}\right]$$

Where a change is missed, the identity of the changed item is guessed at from the set of items not held in memory. The correct identification rate for misses (*MID*) is therefore given by

$$MID=\frac{1}{N-k}$$

Memory capacity is a free parameter.

The HTT model generates linear ROCs which pass through the point 1,1 and have a y-intercept of *k/N*.

#### Noiseless slots with lapses of attention (HTTa)

**Fig 2:**
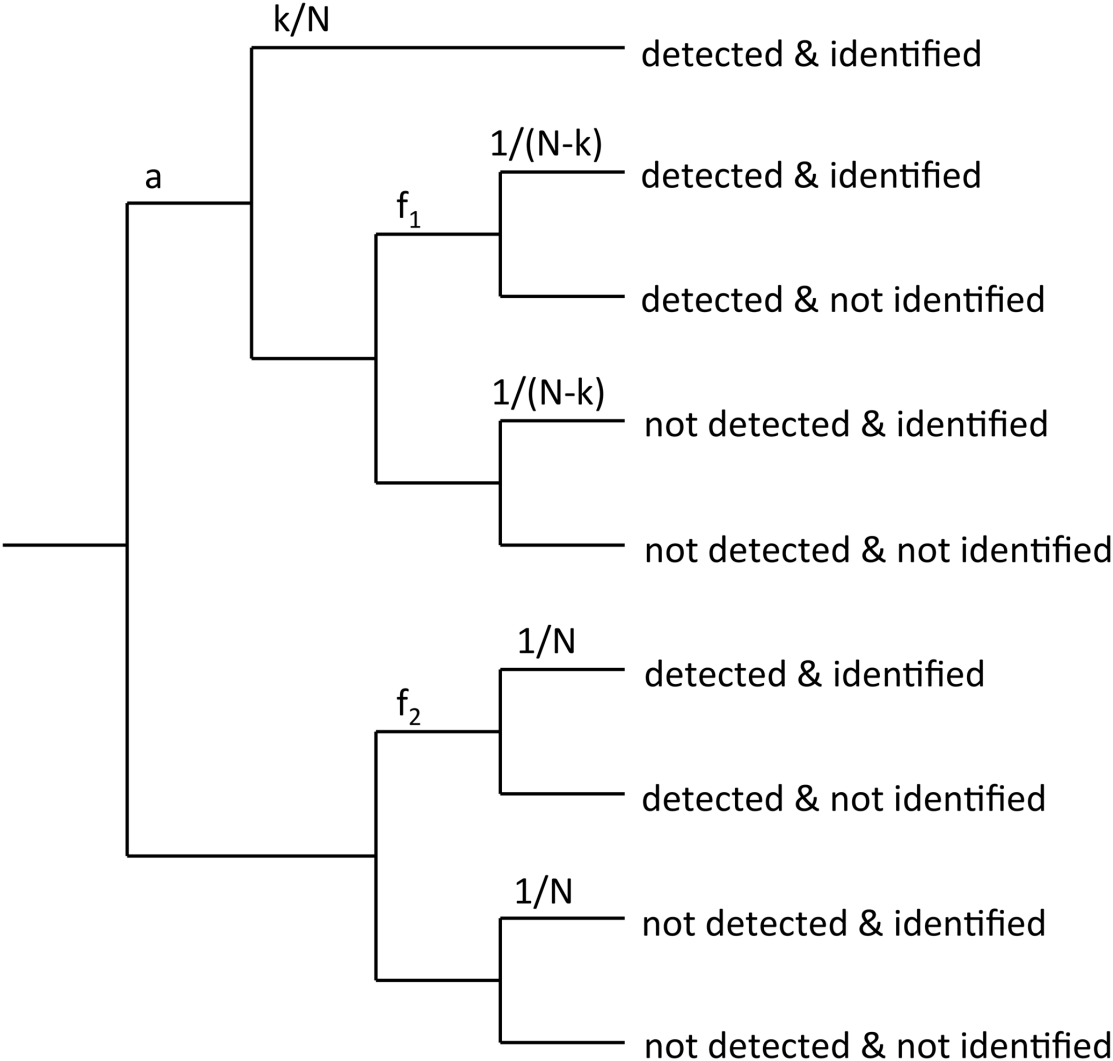
HTTa model. Decision tree for the HTTa model for trials on which a change occurred.

This model is a variant of HTT that allows for lapses of attention [5]. For this model, the observer is attentive on a proportion of trials (*a*) and inattentive on all others. Attended trials are processed according to standard HTT but on unattended trials no information is stored in memory, changes are detected at the false alarm rate for unattended trials, and identity is guessed at from the set of all items (Fig 2).

The total false—alarm rate (*F*) reflects the false—alarm rates on attended trials (*f*_*1*_) and unattended trials (*f*_*2*_), which are both assumed to be proportional to the number of items about which no information is held in memory. It is given by

$$F=af_{1}+\left(1-a\right)f_{2}=af_{1}+\left(1-a\right)\frac{f_{1}N}{N-k}$$

A hit occurs on an attended change trial if, and only if, either of the following two mutually exclusive events occurs: (i) the changed item is held in memory or (ii) the changed item is not held in memory but the observer guesses that there was a change (which he/she does at rate *f*_1_). A hit occurs on an unattended change trial if the observer guesses that there was a change (which he/she does at rate *f*_2_). The hit rate is therefore given by

$$H=a\left[\frac{k}{N}+\left(1-\frac{k}{N}\right)f_{1}\right]+\left(1-a\right)f_{2}$$

Where a hit occurs, the changed item is correctly identified when it is held in memory (which is the case only on attended trials). When it is not held in memory, its identity is guessed at from the set of items not held in memory (which in the case of unattended trials is the set of all items). The correct identification rate for hits is therefore given by

$$HID=\frac{1}{H}\left[a\left(\frac{k}{N}+\frac{\left(1-\frac{k}{N}\right)f_{1}}{N-k}\right)+\left(1-a\right)\frac{f_{2}}{N}\right]$$

Where a change is missed, the identity of the changed item is guessed from the set of items not held in memory. The correct identification rate for misses is therefore given by

$$MID=\frac{1}{1-H}\left[\frac{a\left(1-\frac{k}{N}\right)\left(1-f_{1}\right)}{N-k}+\frac{\left(1-a\right)\left(1-f_{2}\right)}{N}\right]$$

Memory capacity and attention rate are free parameters.

The HTTa model generates linear ROCs which are not constrained to pass through the point 1,1, and have a y-intercept of *ak/N*.

#### Noisy slots (HTTn)

This model is a variant of HTT in which memory representations are limited in both number and precision [10]. For this model, memory representations may be less precise than required to support accurate task performance. In the context of this study, an insufficiently precise representation would be one that results in the incorrect retrieval of a symbol's shape (e.g., the retrieval of a triangle or a rectangle when a semi-circular symbol was displayed). Obviously, a very imprecise representation would be required for that to occur, but this model was included as it is an implementation of a contemporary theory of VSTM [10].

A change to an item represented in memory is correctly detected if, and only if, any of the following four mutually exclusive events occurs: (i) the before-change shape of the changed item is correctly retrieved from memory, (ii) the before-change shape of the changed item is incorrectly retrieved from memory and the retrieved shape differs from the after-change shape of that item, (iii) the before-change shape of the changed item is incorrectly retrieved from memory as the shape that matches the after-change shape of that item but the shape of at least one of the non-changed items is incorrectly retrieved from memory, or (iv) the observer guesses that there was a change when he/she does not perceive a change to any of the items represented in memory (i.e., when the retrieved shapes of all items represented in memory match the shapes of the corresponding items in the second display).

The probability that a change to an item represented in memory is correctly detected is therefore

$$r+\frac{\left(d-2\right)\left(1-r\right)}{d-1}+\frac{1-r}{d-1}\left(1-r^{k-1}\right)+\frac{1-r}{d-1}r^{k-1}t$$

where *r* is the probability that the shape of an item represented in memory is correctly retrieved, *d* is the size of the set of possible item shapes, and *t* is the rate at which the observer reports change when he/she does not perceive a change to any of the items represented in memory.

A change to an item not represented in memory is correctly detected if, and only if, either of the following two mutually exclusive events occurs: (i) the shape of at least one of the items represented in memory is incorrectly retrieved or (ii) the observer guesses that there was a change when he/she does not perceive a change to any of the items represented in memory. The probability that a change to an item not represented in memory is correctly detected is therefore

$$(1-r^{k})+r^{k}t$$

The hit rate is therefore given by

$$H=\frac{k}{N}\left(r+\frac{\left(d-2\right)\left(1-r\right)}{d-1}+\frac{1-r}{d-1}\left(1-r^{k-1}\right)+\frac{1-r}{d-1}r^{k-1}t\right)+\frac{N-k}{N}\left(1-r^{k}+r^{k}t\right)$$

Where a change is perceived, the identity of the changed item is guessed at from the set of items perceived to have changed. Only if a changed item is included in this set, therefore, can its identity be correctly guessed. When it is included in this set, the probability of its identity being correctly guessed depends on the size of this set (*s*). The probability that a changed item is included in the set of items perceived to have changed and that its identity is correctly guessed is

$$\sum_{s=1}^{k}\left(\frac{k}{N}r{\left(1-r\right)}^{s-1}r^{k-s}\frac{\left(k-1\right)!}{\left(s-1\right)!\left(k-s\right)!}\;\frac{1}{s}+\frac{k}{N}\;\frac{\left(d-2\right)\left(1-r\right)}{d-1}{\left(1-r\right)}^{s-1}r^{k-s}\frac{\left(k-1\right)!}{\left(s-1\right)!\left(k-s\right)!}\;\frac{1}{s}\right)$$

$$=\frac{d-2+r}{\left(d-1\right)N}\overset{k}{\underset{s=1}{\sum }}{\left(1-r\right)}^{s-1}r^{k-s}\frac{k!}{s!\left(k-s\right)!}$$

$$=\frac{d-2+r}{\left(d-1\right)N}\;\frac{1-r^{k}}{1-r}$$

Where no change is perceived, the identity of the changed item is guessed at from the set of items not held in memory. Only if a changed item is not held in memory, therefore, can its identity be correctly guessed. The probability that a changed item is not held in memory, no change is perceived but the observer guesses that there was a change, and the identity of the changed item is correctly guessed is

$$\frac{N-k}{N}r^{k}t\frac{1}{N-k}$$

The correct identification rate for hits is therefore given by

$$HID=\frac{1}{H}\left(\frac{d-2+r}{\left(d-1\right)N}\frac{1-r^{k}}{1-r}+\frac{r^{k}t}{N}\right)$$

Where a change is missed, the identity of the changed item is guessed at from the set of items not held in memory. Only if the changed item is not held in memory, therefore, can its identity be correctly guessed. The probability that a changed item is not held in memory, the change is missed, and the identity of the changed item is correctly guessed is

$$\frac{N-k}{N}r^{k}\left(1-t\right)\frac{1}{N-k}$$

The correct identification rate for misses is therefore given by

$$MID=\frac{1}{1-H}\;\frac{r^{k}\left(1-t\right)}{N}$$

Memory capacity and the probability that the shape that an item represented in memory is correctly retrieved are free parameters.

Where the precision of memory representation is always sufficient for the shape of an item to be correctly retrieved (i.e., *r* = 1), the model reduces to the standard HTT model.

The HTTn model produces linear ROCs which pass through the point 1,1 and have a y-intercept equal to

$$\frac{k\left(dr-1\right)r^{k-1}}{\left(d-1\right)N}-r^{k}+1$$

#### Continuous resources (SDT)

Eight independent, noisy change detectors (i.e., one for each symbol on the display) were modelled with unequal-variance SDT. For this model, a change is detected when the activation of at least one change detector exceeds criterion. A false alarm is made when this occurs on a no-change trial. The item associated with the detector with the highest activation is identified as having changed.

According to this model, no change is reported on a no-change trial (i.e., a correct rejection occurs) where the activity in none of the change detectors reaches criterion. The correct-rejection rate (*CR*) is therefore given by

$$CR\;={\left[\emptyset (c)\right]}^{N}$$

Where *ϕ* is the standard normal cumulative distribution function and *c* is the criterion measured in standard deviations of the noise distribution from its the mean.

A miss occurs where the activity in the stimulated change detector fails to reach criterion and the remaining change detectors correctly reject. The miss rate (M) is therefore given by

$$M=\emptyset \left(\frac{c-d\prime }{s}\right){\left[\emptyset \left(c\right)\right]}^{N-1}$$

where *d'* is detector sensitivity measured in standard deviations of the noise distribution, and *s* is the ratio of the standard deviations of the signal-plus-noise and noise distributions.

The HID and MID rates for this model were estimated by Monte Carlo simulation of ten thousand trials for each combination of parameters evaluated during model parameter estimation.

Detector sensitivity and the ratio of the standard deviations of the signal-plus-noise and the noise distributions are free parameters.

This multidimensional SDT model generates asymmetric, nonlinear ROCs which are constrained to pass through the points 0,0 and 1,1. When expressed in z coordinates, the ROCs are linear if the variances of the noise and signal-plus-noise distributions are equal and nonlinear if they are not.

### Model fitting

Each model process was programmed in Matlab (Mathworks) and fitted to data for each observer and condition of change probability. For each model, the free parameters discussed above define its ROC. Nine response biases are additional free parameters that define the points on the model’s ROC that correspond to the observed ROC hit and false-alarm pairs. Maximum likelihood estimates of model parameters were first obtained by minimising the summed deviance from the set of observed hit and false-alarm pairs using a simplex gradient descent algorithm [14]. As ROCs describe change detection performance, models based on these parameter estimates are best fits to the change detection data. We then examined whether the models could parsimoniously predict both change detection and change identification performance. Model parameters were re-estimated by minimising summed deviance from the observed identification rate for hits (HID) and for misses (MID) in addition to the set of observed hit and false-alarm pairs. For all models it was necessary to estimate the average response criterion when fitting identification data. This was done by including the observed false-alarm rate as a fixed parameter.

Models were compared by calculating the Bayes Information Criterion (BIC; [15]) to allow for differences in model flexibility arising from differences in the number of free parameters. The model with the lowest BIC is that with the highest posterior probability given the observed data. If all models are assigned equal prior probability, the posterior probability of each model relative to the most likely model is given by the Bayes factor (BF). Model comparison was conducted for each individual observer.

## Results

Several measures of change detection and identification performance averaged across observers are shown in Table 1 for each of the two conditions of change probability. For both conditions, detection accuracy was close to 80%, the hit rate was around 75%, the false-alarm rate was around 18%, the HID rate was close to 85%, and the MID rate was around 24%. None of these measures differed significantly between the change probability conditions.

**Table 1:** Change detection and identification performance in each condition of change probability (.25 and .75).

	.*25*	.*75*	*t(9)*	*p*
Detection accuracy (%)	80.6 (2.3)	77.4 (3.4)	1.63	.14
Hits (%)	76.0 (3.9)	75.7 (4.2)	0.14	.89
False alarms (%)	17.8 (2.2)	17.7 (3.4)	0.06	.95
Hits correctly identified (%)	83.7 (3.8)	86.6 (3.4)	1.24	.25
Misses correctly identified (%)	23.8 (4.1)	24.1 (1.8)	0.09	.93

Note: Standard errors of the means are shown in parentheses.

**Fig 3:**
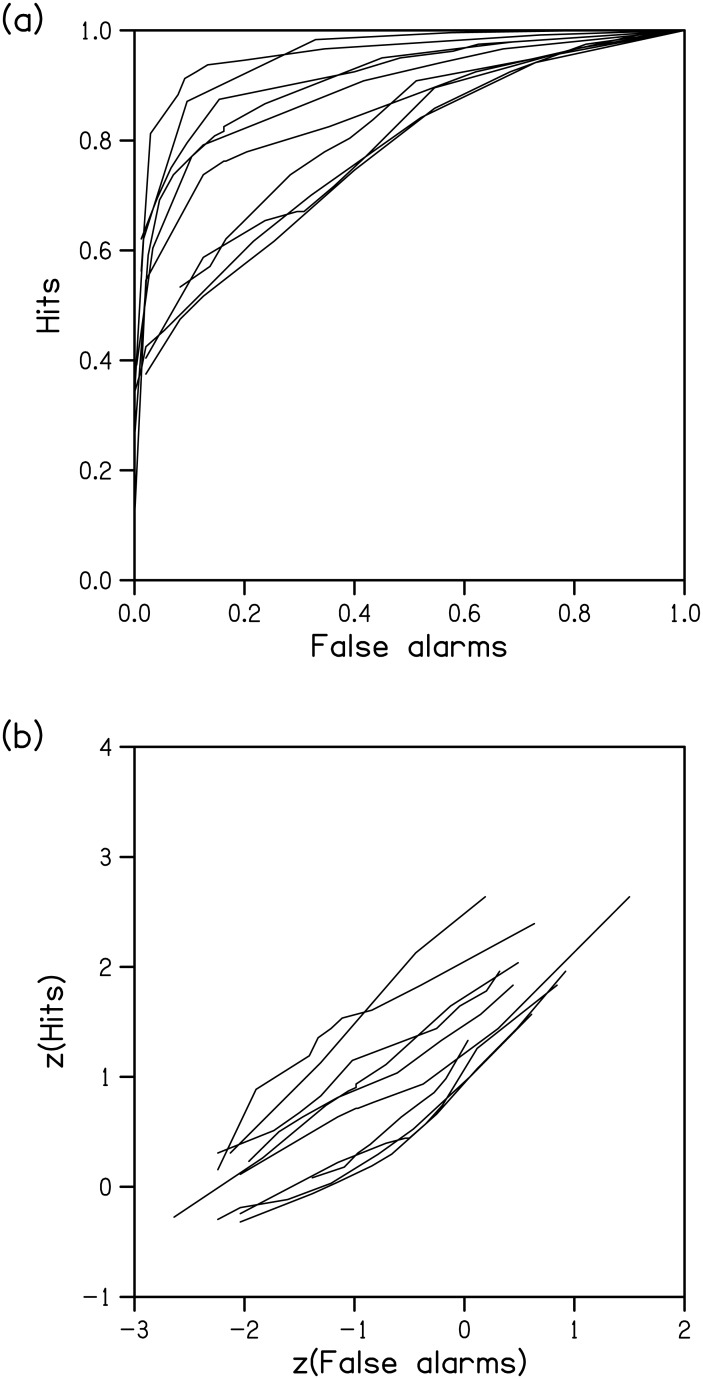
Individual ROCs. ROC for each observer for the change detection task in linear coordinates (a) and z coordinates (b).

ROCs were generated for each observer from his/her detection data (Fig 3a). There was a range of sensitivity across observers, reflected in the differences in the distances of the ROCs from the positive diagonal. Six of the ten ROCs were nonlinear with the regression of hits against false alarms containing a significant quadratic component *t* ≥ 3.1, *p* ≤ .02, which is often considered to be inconsistent with a HTT process [13]. When plotted in z coordinates, six of the ten ROCs (Fig 3b) were nonlinear with a significant quadratic component, *t* ≥ 3.7, *p* ≤ .01. This has been argued to be inconsistent with a purely SDT process [16].

**Fig 4:**
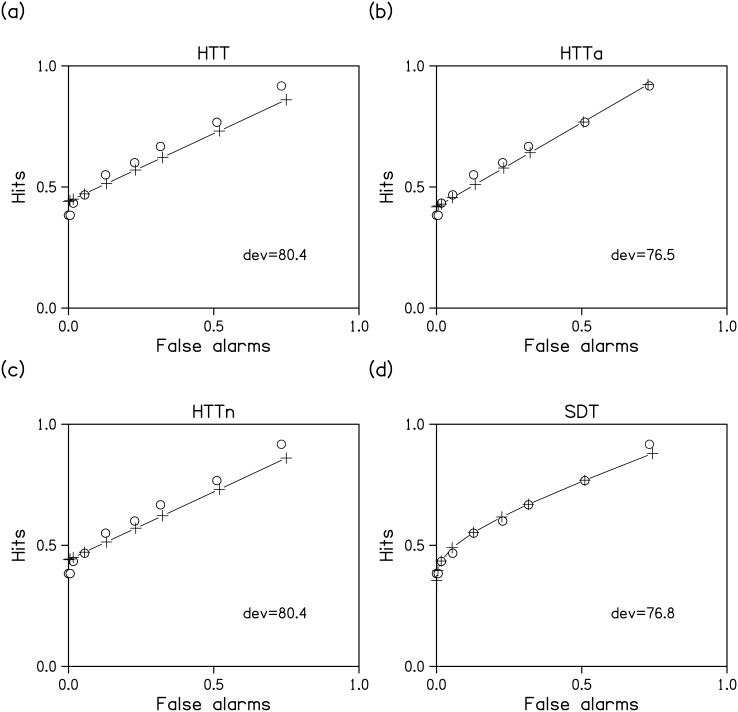
Example model fits. ROC for one observer in the .25 change condition (circles) and the best fitting ROC for each model (crosses). Dev = deviation.

To enable comparison with previous studies of change detection, we first fitted models to detection data only. The fit of each model to the ROC for one observer in the .25 change condition is shown in Fig 4. When model predictions were pooled across observers and conditions of change probability, all models were able to fit the observed ROC points very well. Intraclass correlations for absolute agreement between observed and fitted hits and false alarms are presented in Table 2 for each model. However, rather than formally comparing model fits with regard to these correlations, we compared them with regard to BICs and BFs.

**Table 2:** Intraclass correlations between observed and fitted hits and false alarms for each model.

*Model*	*Hits*	*False alarms*
HTT	.909	.979
HTTa	.947	.972
HTTn	.907	.980
SDT	.999	.999

Note: All correlations are significant, *p* < .001.

For 9/10 observers, the SDT model was the most likely, as indicated by the lowest BIC (Table 3). The evidence favouring the SDT model was strong (BF ratio > 10; [17]) for six observers and moderate (BF ratio > 3) for one (Table 4). There was also moderate evidence favouring the HTTa model for one observer.

**Table 3:** BICs for models fitted to detection data from individual observers.

	*HTT*	*HTTa*	*HTTn*	*SDT*
O1	264.4	239.8	271.6	**235.5**
O2	792.6	647.3	799.8	**177.2**
O3	258.6	247.2	265.8	**245.1**
O4	523.6	472.1	530.8	**224.8**
O5	411.8	317.6	419.0	**232.2**
O6	266.8	252.8	273.9	**252.6**
O7	272.5	**257.0**	279.6	259.4
O8	394.4	375.3	401.6	**224.5**
O9	314.7	272.7	321.9	**231.4**
O10	457.3	369.5	465.1	**227.4**

Note: The smallest BIC for each observer is indicated in bold.

**Table 4:** BFs for models fitted to detection data from individual observers.

	*HTT*	*HTTa*	*HTTn*	*SDT*
O1	.00	.12	.00	**1.00**
O2	.00	.00	.00	**1.00**
O3	.00	.36	.00	1.00
O4	.00	.00	.00	**1.00**
O5	.00	.00	.00	**1.00**
O6	.00	.91	.00	1.00
O7	.00	**1.00**	.00	.30
O8	.00	.00	.00	**1.00**
O9	.00	.00	.00	**1.00**
O10	.00	.00	.00	**1.00**

Note: BFs for models with moderate (BF ratio > 3) or strong (BF ratio > 10) favouring evidence are indicated in bold.

For the nine observers for whom the SDT model was most likely, we examined whether it predicted the observed HID and MID rates. The average predicted HID rate (.92) was close to that observed (.86), but the average predicted MID rate (.55) was much higher than observed (.24). As the SDT model substantially over-predicted MID rates, we included a variant of the SDT model (SDTg) in which the identity of missed changes was sometimes guessed at from the set of all items when fitting models to identification and detection data combined. Whereas the SDT model assumes that the observer always identifies the item associated with the most highly activated detector, the SDTg model assumes he/she adopts this strategy on all trials in which a change is detected, but on only some of the trials in which a change is missed. The SDTg model assumes that on other trials in which a change is missed, the observer chooses to make random guesses at the identities of the changed item without reference to detector activations. For the SDTg model, detector sensitivity, the ratio of standard deviations of the signal-plus-noise and noise distributions, and the rate at which guesses are made at the identities of missed changes (*g*) are free parameters.

For 7/10 observers, one of the two SDT models was the most likely, as indicated by the lowest BIC (Table 5). The evidence favouring the SDT class of model, compared with the HTT class, was strong for six observers (Table 6). There was moderate or strong evidence favouring the SDTg model for four observers and moderate evidence favouring the SDT model for one observer. There was also strong evidence favouring the HTTa model for two observers.

**Table 5:** BICs for models fitted to combined detection and identification data from individual observers.

	*HTT*	*HTTa*	*HTTn*	*SDT*	*SDTg*
O1	294.3	269.4	301.7	**268.3**	270.8
O2	830.0	745.6	826.6	266.9	**218.5**
O3	291.2	**269.8**	298.3	277.4	283.8
O4	552.4	532.5	555.8	257.6	**256.1**
O5	448.2	368.0	454.4	272.3	**266.4**
O6	339.0	**284.0**	327.5	310.4	310.7
O7	301.4	**285.9**	308.0	287.6	293.2
O8	454.3	425.1	451.2	265.2	**255.7**
O9	361.8	280.9	365.3	**266.6**	269.7
O10	507.1	405.6	497.8	285.1	**281.6**

Note: The smallest BIC for each observer is indicated in bold.

**Table 6:** BFs for models fitted to combined detection and identification data from individual observers.

	*HTT*	*HTTa*	*HTTn*	*SDT*	*SDTg*
O1	.00	.60	.00	1.00	.30
O2	.00	.00	.00	.00	**1.00**
O3	.00	**1.00**	.00	.02	.00
O4	.00	.00	.00	.46	1.00
O5	.00	.00	.00	.05	**1.00**
O6	.00	**1.00**	.00	.00	.00
O7	.00	1.00	.00	.43	.03
O8	.00	.00	.00	.01	**1.00**
O9	.00	.00	.00	**1.00**	.21
O10	.00	.00	.00	.17	**1.00**

Note: BFs for models with moderate (BF ratio > 3) or strong (BF ratio > 10) favouring evidence are indicated in bold.

Parameter estimates for the most likely model (when fitted to the combination of change detection and identification data) for each observer and condition of change probability are shown in Table 7. For each observer, there is broad agreement across the two conditions of change probability with respect to the estimated parameter values. For observer 6, capacity estimates are somewhat higher than typically reported and estimated attention rates are rather low.

**Table 7:** Best fitting parameters for the best fitting model for the combination of detection and identification data for each observer and condition of change probability.

*Observer*	*Model*	*p(change)*	*k*	*a*	*d'*	*s*	*g*
O1	SDT	.25	-	-	2.1	4.3	-
		.75	-	-	2.2	2.4	-
O2	SDTg	.25	-	-	3.0	0.9	.81
		.75	-	-	2.6	0.4	1.0
O3	HTTa	.25	4.8	.69	-	-	-
		.75	4.1	.80	-	-	-
O4	SDTg	.25	-	-	3.1	1.6	.93
		.75	-	-	3.0	1.8	.75
O5	SDTg	.25	-	-	3.0	1.1	.77
		.75	-	-	3.1	1.3	.74
O6	HTTa	.25	8.0	.40	-	-	-
		.75	5.9	.66	-	-	-
O7	HTTa	.25	5.0	.83	-	-	-
		.75	4.6	.73	-	-	-
O8	SDTg	.25	-	-	3.7	0.9	1.0
		.75	-	-	3.4	1.0	.73
O9	SDT	.25	-	-	2.9	1.3	-
		.75	-	-	3.8	1.7	-
O10	SDTg	.25	-	-	3.2	1.9	.02
		.75	-	-	3.0	1.3	.71

## Discussion

The present study found that of the models tested to account for change detection performance in isolation, that based on SDT was supported by the evidence for most (i.e., 7/10) of the observers. This model assumed that task performance was supported by a continuous memory resource that was distributed across all items in our displays to form eight, noisy representations. The SDT class of model was in the majority of cases (i.e., 6/10) also able to account for change identification performance. For some observers, an additional parameter to allow for guessing at the identity of missed changes was required because the standard SDT model over-predicted the MID rate. According to SDT, the relative activation of detectors carries information about the identity of a change, even when the activation does not reach criterion for detection (i.e., when a change is missed). It is presumed that some observers adopted a strategy on some miss trials that did not take advantage of this information.

For a small number observers (i.e., 1/10 in the case of fitting models to detection data in isolation and 2/10 in the case of fitting them to detection and identification data combined), however, it was found that the evidence supported the HTT-based model that allowed for lapses of attention. This model assumed that task performance was supported by a discrete memory resource capable of maintaining a limited number of noiseless representations.

Although it is possible, it seems unlikely that the fundamental architecture of short-term memory differs across individuals. The inconsistency across observers in our study with regard to preferred model, therefore, more probably stemmed from other inter-observer differences. One possibility is that some observers made use of verbal short-term memory to form robust (i.e., HTT-like) representations of a subset of the items in our displays. Capacity limitations associated with this memory system, such as those imposed by the time required to recode visual information into a verbal form, may have restricted the number of items that could be represented verbally to fewer than eight.

A second possibility is that the three-second display inspection time in our study was too short for some observers to fixate on each of the eight items. Although eye movements were not measured, it is clear that most observers inspected the items in our displays in a serial fashion, fixating briefly on each. It is likely that fixated and non-fixated items would be processed to different extents and represented in memory with vastly different degrees of robustness. In tests conducted subsequent to this study, in which eye movements were measured, we found that three additional observers fixated on an average of 7.8 items while performing our change detection and identification task. This suggests that a three-second inspection time is adequate for observers to fixate on all eight items on most trials, but it remains possible that it is inadequate for some. Evidence favouring a HTT- over a SDT-based model of VSTM was previously reported in a study where observers were allowed to move their eyes while they inspected the initial display in a change-detection paradigm but restricted inspection time to 500 ms [5]. An inspection time of this duration would have provided sufficient time for observers to make one or two saccades [18], and thus fixate on some but not all items except in the smallest array size condition.

We cannot exclude the possibility that observers with ROCs that were well fitted by SDT also engaged in verbal labelling. Recognition memory for words is commonly modelled using SDT [16]. In the Introduction, we suggested that item representations based on verbal labels may be more robust than those held in VSTM. Verbally based representations, however, need not necessarily be entirely noiseless, and it is possible that they are sometimes well modelled by SDT. If some of the observers in our study held some display items in verbal memory and others in VSTM, their detection performance may reflect that of an array of detectors of differing sensitivity. It is possible that the performance of such an array would be well modelled by a standard (i.e., fixed detector sensitivity) unequal-variance SDT model that accommodates the variance in actual detector sensitivity in the model signal-plus-noise variance. We confirmed this by simulating the performance of an eight-detector array in which four detectors had a *d'* of 1 and the other four had a *d'* of 3. The standard deviation of the noise in all detectors was set to 1. An unequal-variance SDT model with a *d'* of 2 and a signal-plus-noise standard deviation of 1.7 was found to provide an excellent fit to the ROC of this mixed sensitivity array (deviance = 59.6, which is similar to the smallest deviances observed in this study). The corollary of this result is that previous studies that have found evidence for multidimensional unequal-variance SDT could alternatively be interpreted to have found evidence for a mixture of detector sensitivities across items, which may reflect differences in levels of processing across those items.

In conclusion, the present study has extended on previous studies by examining visual change detection and identification performance under free viewing conditions akin to those usually present in real-world settings. The visual eccentricity of items was not constrained and the stimulus duration allowed serial fixation. We found that for the majority of observers, both change detection and identification performance could be parsimoniously described by a single underlying SDT-based process. However, for a small number of observers, there was strong evidence in support of a HTT-based model that allowed for lapses of attention.
